# Adipose tissue and metabolic and inflammatory responses to stroke are altered in obese mice

**DOI:** 10.1242/dmm.030411

**Published:** 2017-10-01

**Authors:** Michael J. Haley, Graham Mullard, Katherine A. Hollywood, Garth J. Cooper, Warwick B. Dunn, Catherine B. Lawrence

**Affiliations:** 1 Faculty of Biological, Medical and Human Sciences, Manchester Academic Health Science Centre, University of Manchester, Manchester, M13 9PT, UK; 2 Centre for Endocrinology and Diabetes, Institute of Human Development, Faculty of Biological, Medical and Human Sciences, University of Manchester, Manchester, M13 9WL, UK; 3 Centre for Advanced Discovery and Experimental Therapeutics (CADET), Central Manchester University Hospitals NHS Foundation Trust, Manchester Academic Health Sciences Centre, Manchester, M13 9WL, UK; 4 School of Chemistry, Manchester Institute of Biotechnology, University of Manchester, 131 Princess Street, Manchester, M1 7DN, UK; 5 Maurice Wilkins Centre for Molecular Biodiscovery, Faculty of Science, University of Auckland, Auckland 1020, New Zealand; 6 Department of Pharmacology, University of Oxford, Mansfield Road, Oxford, OX1 3QT, UK; 7 School of Biosciences and Phenome Centre Birmingham, University of Birmingham, Edgbaston, Birmingham, B15 2TT, UK

**Keywords:** Stroke, Metabolomics, Obesity, Lipids, Inflammation, Adipokines

## Abstract

Obesity is an independent risk factor for stroke, although several clinical studies have reported that obesity improves stroke outcome. Obesity is hypothesised to aid recovery by protecting against post-stroke catabolism. We therefore assessed whether obese mice had an altered metabolic and inflammatory response to stroke. Obese *ob/ob* mice underwent a 20-min middle cerebral artery occlusion and 24-h reperfusion. Lipid metabolism and expression of inflammatory cytokines were assessed in the plasma, liver and adipose tissue. The obese-specific metabolic response to stroke was assessed in plasma using non-targeted ultra-high performance liquid chromatography-mass spectrometry (UHPLC-MS) metabolomics coupled with univariate and multivariate analysis. Obesity had no effect on the extent of weight loss 24 h after stroke but affected the metabolic and inflammatory responses to stroke, predominantly affecting lipid metabolism. Specifically, obese mice had increases in plasma free fatty acids and expression of adipose lipolytic enzymes. Metabolomics identified several classes of metabolites affected by stroke in obese mice, including fatty acids and membrane lipids (glycerophospholipids, lysophospholipids and sphingolipids). Obesity also featured increases in inflammatory cytokines in the plasma and adipose tissue. Overall, these results demonstrate that obesity affected the acute metabolic and inflammatory response to stroke and suggest a potential role for adipose tissue in this effect. These findings could have implications for longer-term recovery and also further highlight the importance of considering comorbidities in preclinical stroke research, especially when identifying biomarkers for stroke. However, further work is required to assess whether these changes translate into long-term effects on recovery.

## Abstract (precis)

**Summary:** Obesity, a co-morbidity for stroke, affected the acute metabolic and inflammatory response to stroke, highlighting the importance of considering comorbidities in preclinical stroke research, especially when identifying biomarkers.

## INTRODUCTION

Obesity is a major health problem worldwide that is characterised by excessive fat accumulation, disruption of normal metabolic processes and a state of chronic low-level inflammation. During obesity, adipocytes become stressed due to their energy storage capacity being exceeded, leading to adipose tissue inflammation. Rather than being simply a passive energy store, adipose tissue can release a wide variety of signalling molecules termed adipokines. Inflammation within the adipose tissue therefore leads to the release of pro-inflammatory mediators and adipokines into the circulation. Besides having an established role in immune regulation and energy balance, adipokines have been found to regulate an expanding array of physiological functions, including haemostasis, lipid and glucose metabolism, blood pressure, insulin sensitivity, and angiogenesis (Guri and Bassaganya-Riera, 2010; Trayhurn and Wood, 2005). Alterations in adipokine release may therefore affect the function of other organs important for metabolism, for example the liver.

Although being obese can result in several conditions that are associated with increased stroke risk, including hypertension, diabetes and high cholesterol, obesity has now been identified as an independent risk factor for stroke (Strazzullo et al., 2010). However, some epidemiological studies in patients have reported a protective effect of obesity on stroke outcome, including reduced long-term mortality and improved functional recovery (Doehner et al., 2013; Vemmos et al., 2011). This so-called ‘obesity paradox’ may potentially be explained by design of the epidemiological studies, including complications arising from how obesity is measured, age, type and severity of stroke, and study design (Haley and Lawrence, 2016a). A biological hypothesis that has been proposed to explain the obesity paradox is that obesity may protect against the metabolic consequences of stroke (Scherbakov et al., 2011).

Weight loss after stroke is a common clinical observation and metabolic status post-stroke is an important determinant of outcome, with undernourishment worsening functional recovery and survival (FOOD Trial Collaboration, 2003). Impaired feeding behaviour and inactivity due to post-stroke paresis are thought to contribute to post-stroke weight loss, and recommendations for dietary support of patients have been established (Lim and Choue, 2013). However, in both patients and animals, stroke may also have more immediate and direct effects on energy balance via other more rapid mechanisms. Stroke results in an inflammatory response within the first 24 h at the site of ischaemia, systemically and within peripheral organs (Denes et al., 2010a). This response involves induction of inflammatory cytokines such as interleukin-6 (IL-6), and the chemokines C-C motif chemokine 2 (CCL2; MCP-1) and C-X-C motif chemokine 1 (CXCL1; KC). Inflammatory cytokines have been implicated in the pathogenesis of cachexia, a condition featuring loss of lean and adipose tissue weight due to hypermetabolism (Kotler, 2000). Activation of the hypothalamic-pituitary-adrenal (HPA) axis is also a feature of stroke that may promote weight loss through enhanced sympathetic signalling (Fassbender et al., 1994; Mitchell, 1997). The resulting increase in catabolic drive can lead to a loss of adipose and muscle mass, potentially leading to a worse functional outcome due to a loss of muscle function in patients (Scherbakov and Doehner, 2011). Similar losses of muscle mass after stroke have been reported in mice (Desgeorges et al., 2015; Springer et al., 2014). Changes in metabolism may therefore have an important role to play post-stroke and potentially impact pathophysiology and outcome.

Despite the importance of metabolic status in patient outcome, the metabolic response to stroke has undergone little preclinical or clinical investigation. Therefore, the study of global metabolism to understand metabolic differences associated with stroke and the influence of obesity on outcome is an important research area. Metabolomics is the holistic study of low molecular weight chemicals (metabolites) in biological systems. Metabolites are important biochemicals because they act as precursors for the synthesis of other biochemical (e.g. proteins, RNA, DNA) and cellular structures (e.g. cell walls). Because metabolites are the substrates and by-products of cellular metabolism, the quantitative collection of metabolites (defined as the metabolome) provides a sensitive and dynamic phenotypic snapshot of human health and disease (Dunn et al., 2011a; Patti et al., 2012). This phenotypic snapshot can be applied to understand pathophysiological processes or to identify biomarkers associated with disease risk, onset, progression and treatment. Recent studies in animal models and humans have applied metabolomic approaches to understand the molecular pathophysiology of stroke and have highlighted specific important areas of metabolism. These include specific changes in amino acids, fatty acids, sphingolipids, and lysophospholipid species (Gao et al., 2013; Jové et al., 2015; Kimberly et al., 2013; Liu et al., 2015; Sheth et al., 2015; Zhu et al., 2015).

The aim of this study was to assess how the acute metabolic and inflammatory responses to stroke were affected by obesity. In obese mice prior to and 24 h post-stroke, we assessed lipid metabolism and the expression of inflammatory mediators in metabolically active organs. The plasma metabolite profile was then assessed using non-targeted ultra-high performance liquid chromatography-mass spectrometry (UHPLC-MS) combined with univariate and multivariate analysis, allowing the metabolic response to stroke in obese mice to be identified.

## RESULTS

### Stroke alters lipid metabolism in obese mice

**Fig. 1.**
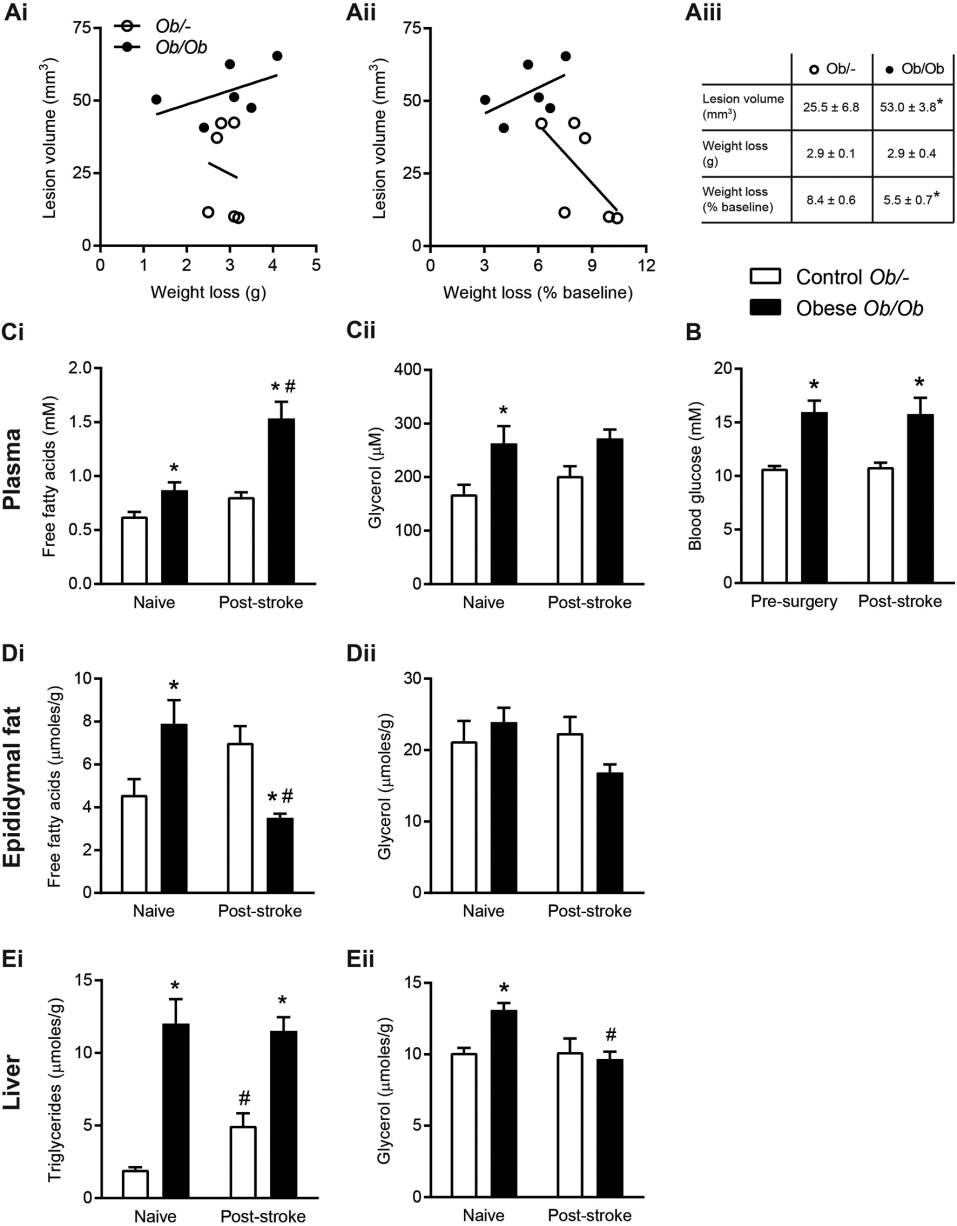
**Stroke had differential effects on fatty acid metabolism in obese (*ob/ob*) and control (*ob/–*) mice.** (A) No significant correlation between body weight loss from the day of surgery to 24 h post-stroke and ischaemic infarct volume was found. (B) Blood glucose was measured in tail vein blood pre-surgery and at 24 h post-stroke. (C,D) In naïve animals and at 24 h post-stroke, free fatty acid (FFA) and glycerol concentrations were measured in plasma from cardiac blood (C) and epididymal fat homogenates (D). (E) Triglyceride and glycerol measurements were made in liver homogenates. Data are means±s.e.m., *n*=10 for naïve, *n*=14 for stroke group, *n*=6 for lesion volume analysis, *P<0.05 versus control *ob/–* for same treatment, ^#^*P*<0.05 versus pre-surgery or naïve for same genotype using (A) Student's *t*-test or (B-E) two-way ANOVA with Šídák correction for multiple comparisons.

Both control *ob/–* and obese *ob/ob* mice lost a significant amount of body weight at 24 h after stroke, but there was no difference in the absolute weight loss between groups (Fig. 1A). However, when expressed as a percentage of pre-surgery weight, *ob/ob* mice lost significantly less weight. In both genotypes, no significant correlation was found between ischaemic damage and either absolute or percentage weight loss. Stroke resulted in significantly greater (108%) ischaemic damage in obese *ob/ob* compared to *ob/–* mice.

Blood glucose levels were significantly increased in obese *ob/ob* compared to control *ob/–* mice prior to surgery, and at 24 h post-stroke. Blood glucose concentrations were not affected by stroke in either control *ob/–* or obese *ob/ob* mice as no significant difference was observed between blood glucose concentrations at 24 h post-stroke when compared to pre-surgery values for obese *ob/ob* and control *ob/–* separately (Fig. 1B).

Obesity *per se* was associated with an increase in plasma free fatty acids (FFAs), as concentrations were significantly greater in naïve *ob/ob* mice. Stroke then resulted in a further increase in plasma FFA in *ob/ob* mice, with concentrations in obese mice being significantly increased relative to both naïve obese mice and control mice post-stroke (Fig. 1Ci). Plasma glycerol was significantly increased in naïve obese *ob/ob* mice, but there was no effect of stroke in either control or obese mice (Fig. 1Cii).

In the epididymal adipose tissue of obese *ob/ob* mice, levels of FFAs were elevated in naïve mice, although, after stroke, there was a significant reduction in adipose FFA in obese *ob/ob* mice relative to controls (Fig. 1Di). No effect of genotype or treatment was observed in glycerol content in adipose tissue (Fig. 1Dii).

Liver triglycerides were significantly higher in naïve *ob/ob* mice compared to control *ob/–* mice, and remained significantly higher after stroke (Fig. 1Ei). However, stroke resulted in a significant increase in liver triglycerides in control *ob/–* mice only. Glycerol levels in the liver were significantly higher in naïve obese *ob/ob* mice. In response to stroke there was a significant reduction in liver glycerol in obese *ob/ob* mice but no change in control mice (Fig. 1Eii).

### Obesity differentially affects expression of adipose lipolytic enzymes

**Fig. 2.**
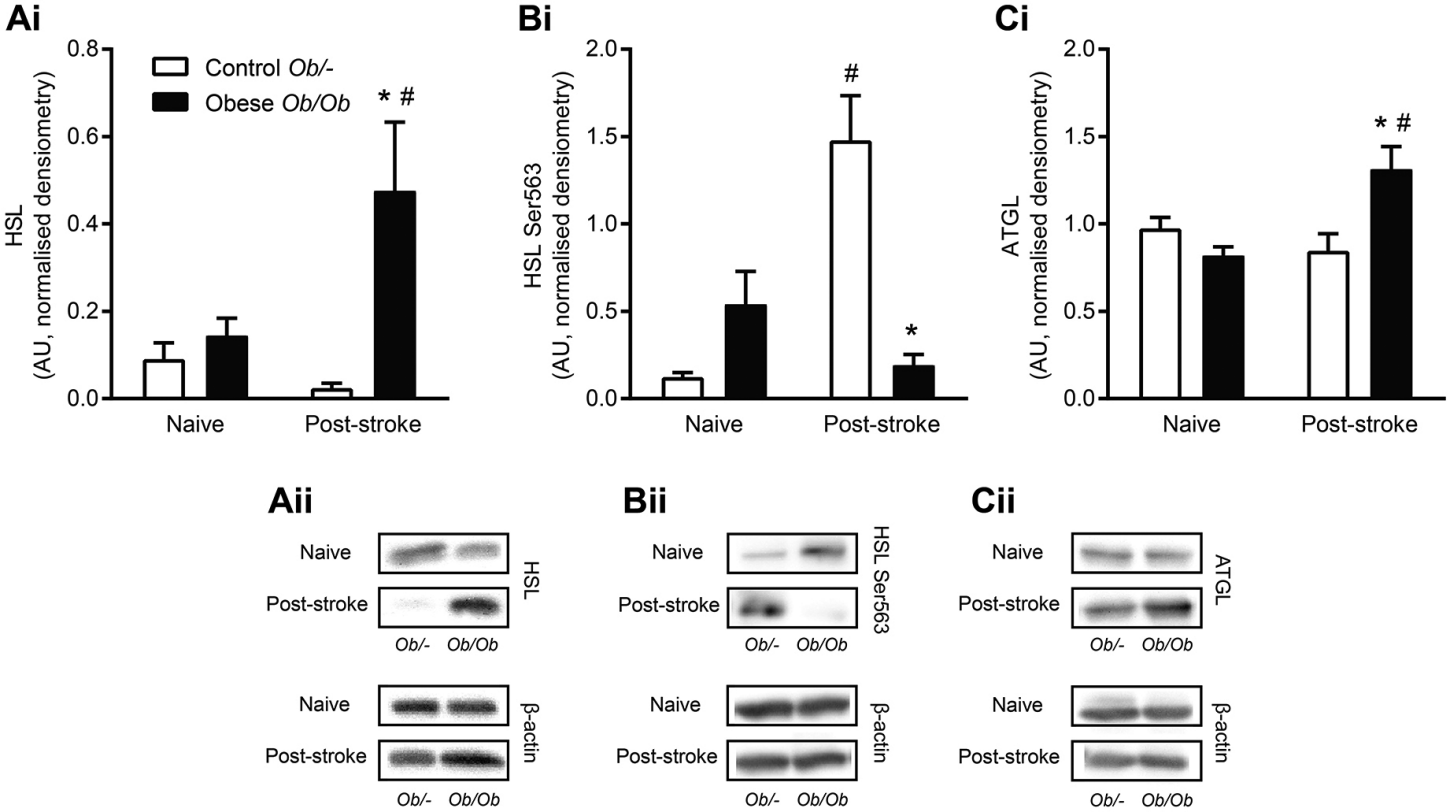
**Obesity differentially affected the expression of the lipolytic enzymes HSL and ATGL in epididymal adipose tissue after stroke.** Expression of hormone sensitive lipase (HSL) (A), serine-563-phosphorylated HSL (B) and adipose triglyceride lipase (ATGL) (C) was quantified by western blot in epididymal adipose tissue homogenates in naïve control *ob/–* and obese *ob/ob* animals, or at 24 h post-stroke. Data are means±s.e.m., *n*=10 for naïve and *n*=14 for stroke groups, *P<0.05 versus control *ob/–* for same treatment, ^#^*P*<0.05 versus naïve for same genotype using two-way ANOVA with Šídák correction for multiple comparisons. Densitometry values were normalised to β-actin as a loading control.

The epididymal adipose tissue expression of the lipolytic enzymes adipose triglyceride lipase (ATGL), hormone-sensitive lipase (HSL) and the Ser563-phosphorylated form of HSL were all similar between naïve control *ob/–* and obese *ob/ob* mice (Fig. 2). HSL expression in obese *ob/ob* mice was significantly increased 24 h after stroke, but remained unchanged in response to stroke in control *ob/–* mice (Fig. 2A). Conversely, expression of HSL Ser563 was significantly increased post-stroke in control *ob/–* mice, with no effect of stroke seen in obese *ob/ob* mice (Fig. 2B). The expression of ATGL was increased in adipose tissue 24 h post-stroke in obese *ob/ob* compared to naïve obese, but was unaffected by stroke in control *ob/–* mice (Fig. 2C).

### Obesity alters the peripheral inflammatory response to stroke in the plasma, epididymal fat and liver

**Fig. 3.**
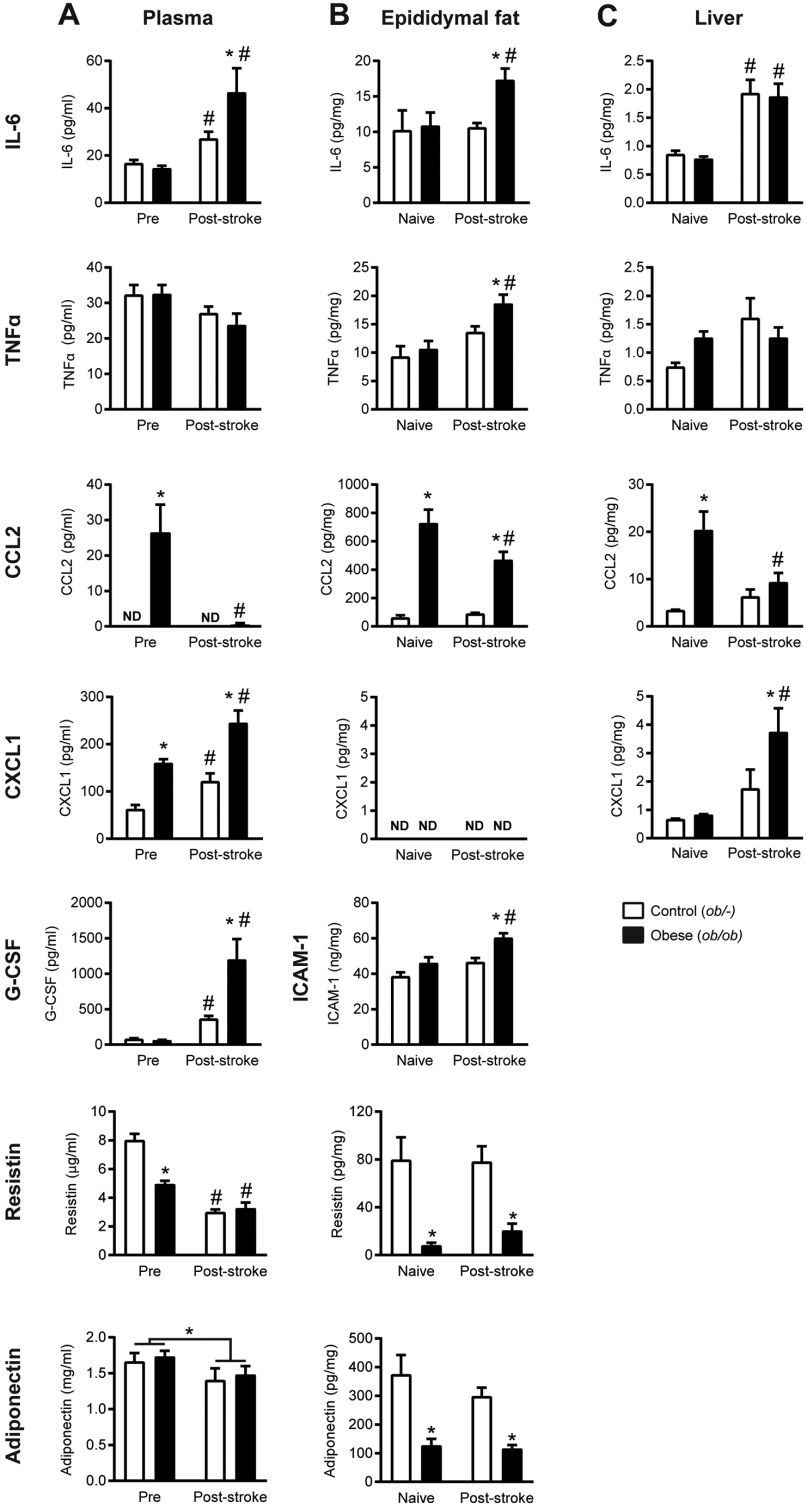
**Obesity altered the expression of inflammatory mediators and adipokines in the plasma and metabolically active organs after stroke.** Measurements were made in plasma from tail vein blood immediately prior to surgery (Pre) and at 24 h post-stroke (A). Measurements were also made in epididymal fat pad (B) and liver (C) homogenates taken from naïve animals or at 24 h post-stroke, and normalised to milligram of total protein as measured by bicinchoninic acid (BCA) assay. Cytokine (IL-6, TNFα, CCL2, CXCL1, G-CSF) concentrations were measured by CBA. ICAM-1, adiponectin and resistin concentrations were measured by ELISA. Data are means±s.e.m., *n*=10 for naïve and *n*=14 for stroke groups, *P<0.05 versus control *ob/–* for same treatment, ^#^*P*<0.05 versus pre-surgery or naïve for same genotype using two-way ANOVA with Šídák correction for multiple comparisons. ND, not detected.

Ischaemic stroke increased expression of some inflammatory mediators, as measured by cytometric bead array in the plasma, epididymal fat and liver (Fig. 3), and this increase was often greater in obese mice. In response to stroke, both obese *ob/ob* and control *ob/–* mice had significantly increased concentrations of plasma and liver IL-6, and plasma granulocyte-colony stimulating factor (G-CSF) and CXCL1 (Fig. 3A,C). However, there were significantly greater responses in obese mice in plasma IL-6, G-CSF and CXCL1, and liver CXCL1 increased in obese mice only. Furthermore, only obese mice showed an increase in inflammatory mediators in the adipose tissue after stroke (Fig. 3B). Obese mice showed increased expression of inflammatory mediators (IL-6, TNFα, ICAM-1) in the epididymal fat after ischaemic stroke, whereas there was no significant effect in control mice. Expression of CCL2 was significantly greater in the plasma, epididymal fat and liver of obese mice prior to stroke compared with control mice. However, stroke then resulted in a significant decrease in CCL2 expression in the plasma and both tissues.

### Stroke reduced plasma concentrations of the adipokines resistin and adiponectin

The expression of the adipokines resistin and adiponectin were measured in the plasma and epididymal adipose tissue by ELISA (Fig. 3). In the epididymal adipose tissue in both naïve mice and post-stroke, obese *ob/ob* mice had significantly lower expression of both resistin and adiponectin compared with control mice (Fig. 3B). Similarly, resistin concentrations were lower in the plasma of naïve obese mice. Stroke then resulted in a significant decrease in plasma concentrations of both resistin and adiponectin (Fig. 3A). Post-stroke plasma concentrations of resistin were significantly lower in both genotypes, and there was a significant effect of treatment (pre- versus post-stroke) on adiponectin concentrations.

### Untargeted metabolomics identified the obese-specific metabolic response to stroke

Samples were randomly analysed applying a UHPLC-MS platform, appropriate for the detection of a wide range of semi-hydrophilic and lipophilic metabolites. To calculate the quality of data acquired and to enable quality assurance of the data, a single quality control (QC) sample was intermittently analysed every 6th injection throughout the run. All metabolite features with a relative standard deviation (RSD) >20% and where there was greater than 30% missing values were removed from the dataset prior to data analysis. This process removes poor quality data and 4891 and 3013 metabolite features remained in positive and negative ion mode, respectively, post-filtering.

**Fig. 4.**
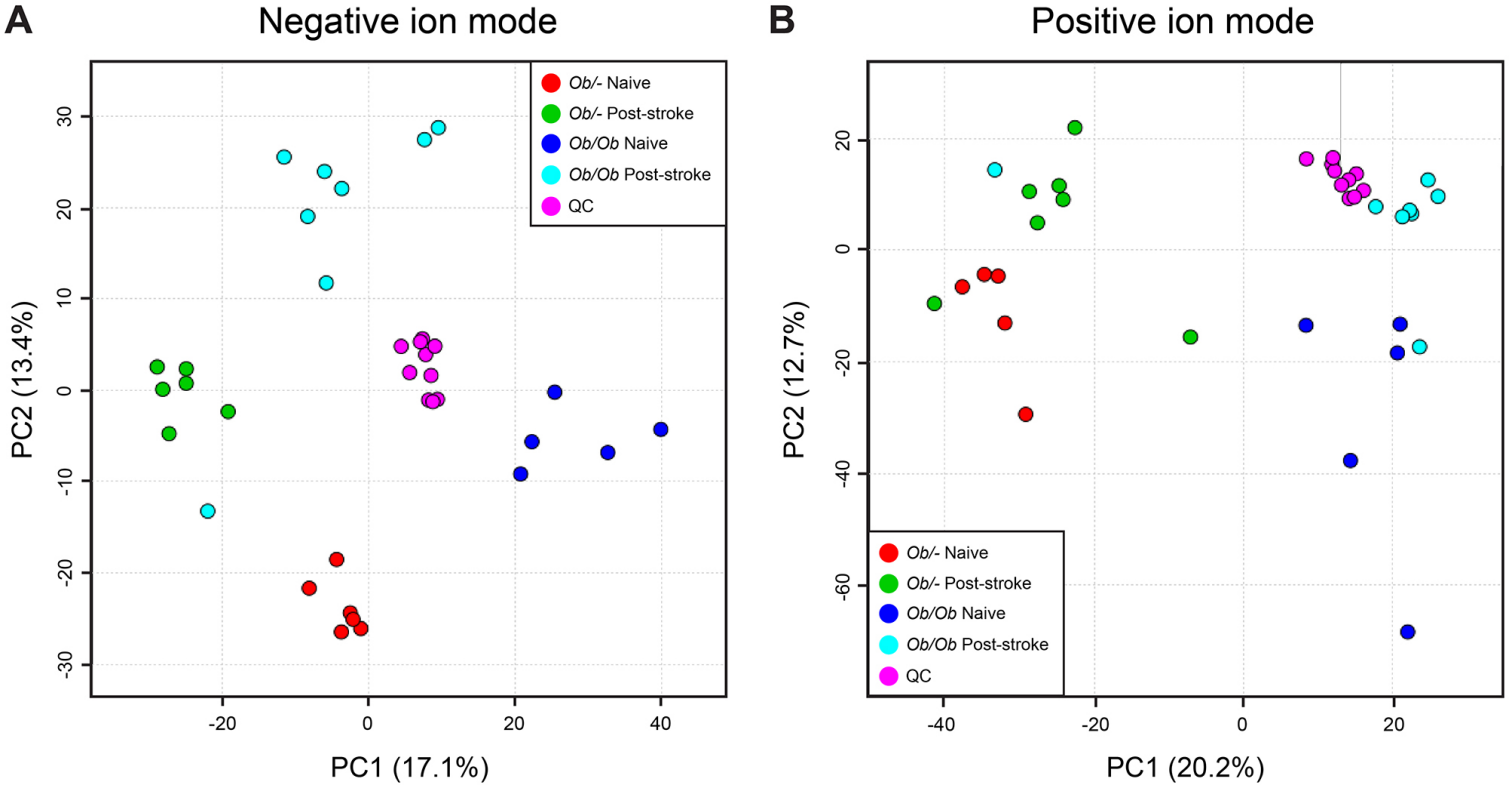
**Principal component analysis (PCA) plots for metabolomics data generated using UHPLC-MS in negative and positive ion modes.** (A) Negative ion mode; (B) positive ion mode. Analysis was performed on plasma 24 h after stroke or in naïve control (*ob/–*) and obese (*ob/ob*) mice. *n*=5-8 animals per group. QC, quality control. Data on class of metabolites is presented in Fig. 5.

Principal components analysis (PCA) was performed to assess the natural variation in the dataset (Fig. 4). The QC samples were tightly clustered compared to the biological samples, highlighting the reproducibility of the data. The scores plot for negative ion mode data (Fig. 4A) showed a separation in principal component 2 (PC2) related to time; samples from naïve control *ob/–* and obese *ob/ob* mice were separated from samples from control *ob/–* and obese *ob/ob* mice 24 h post-stroke. For positive ion mode data (Fig. 4B), separation in PC1 based on control *ob/–* versus obese *ob/ob* animals was suspected, although one sample in the obese 24 h post-stroke class was different to all other samples in the class, which masked this observation.

Supervised partial least squares-discriminant analysis (PLS-DA) was performed to identify multivariate groups of metabolites that discriminate between *ob/–* and *ob/ob* mice in naïve animals, and at 24 h post-stroke. Although validated models were observed when comparing control and obese animals pre-stroke (naïve), validated models were not constructed when comparing control and obese animals 24 h after stroke. Therefore, further biological analysis of the variable importance in projection loadings was not performed.

**Fig. 5.**
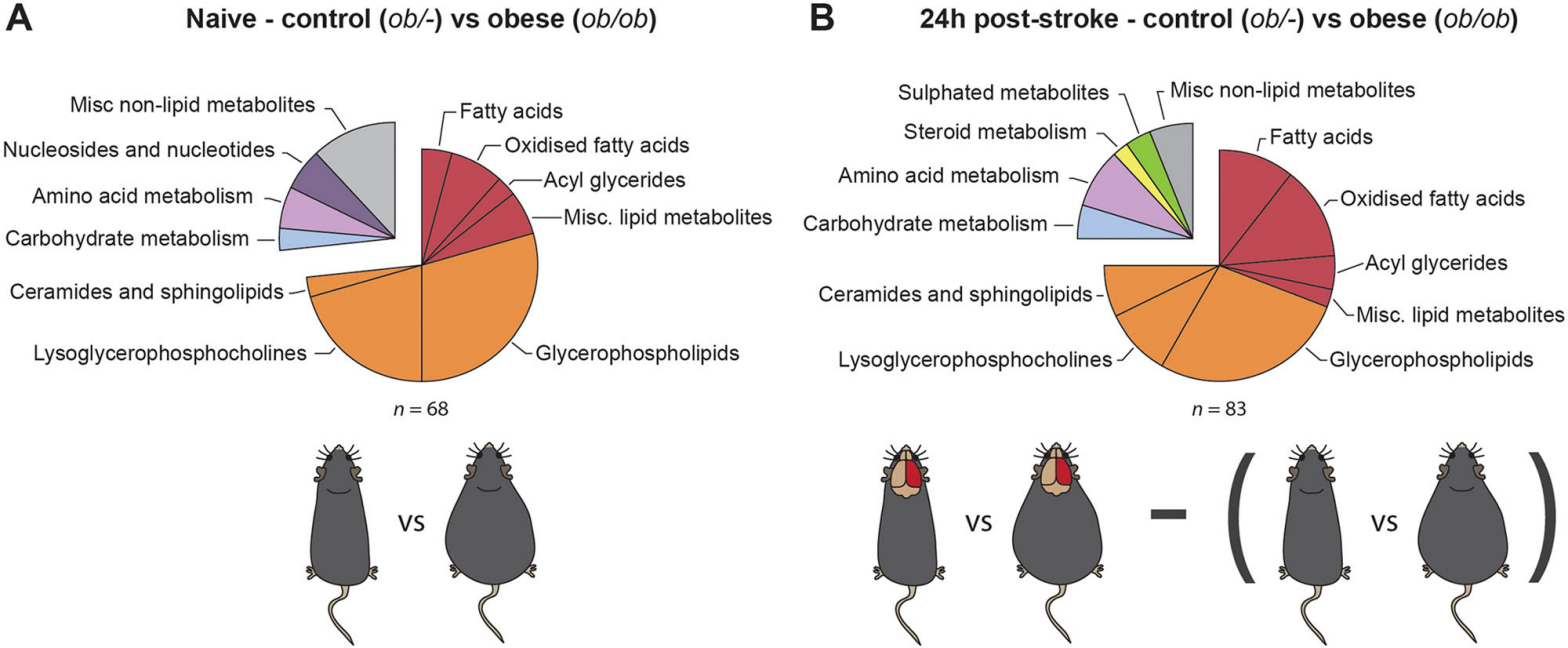
**Obesity affected the metabolic response to stroke.** Classification of plasma metabolites that were significantly different between control (*ob/–*) and obese (*ob/ob*) mice in naïve mice (A) and at 24 h post-stroke (B). Any metabolites that were significantly affected by obesity in the naïve comparison were excluded from the list of metabolites affected by obesity in the 24 h post-stroke comparison. *n*=5-8 animals per group.

Statistical analysis was performed to compare naïve control (*ob/–*) versus naïve obese (*ob/ob*) mice, and control versus obese animals 24 h post-stroke. False discovery rate (FDR) calculations were performed following the Benjamini–Hochberg procedure to limit FDR observations. A total of 68 unique metabolites were observed to be statistically significant when naïve control mice were compared to naïve obese mice (Fig. 5A). Changes in amino acids, fatty acids, oxidised fatty acids, nucleotides, glycerophospholipids and lysophospholipids were observed, and these results indicate metabolic changes related to obesity in the absence of stroke (Table 1). We then compared the metabolic profiles of control (*ob/–*) and obese (*ob/ob*) mice 24 h after stroke. Metabolites that were already found to be significantly altered by obesity in naïve animals were excluded from this list, and so the resulting metabolite profile is the obese-specific metabolic response to stroke. A total of 83 unique metabolites, not observed to be statistically significant between naïve control and obese mice, were found to be statistically significant when comparing control to obese mice 24 h after stroke (Fig. 5B). These metabolites included changes in amino acids, fatty acids, oxidised fatty acids, carbohydrates, ceramides and sphingolipids, sulphated metabolites, glycerophospholipids, and lysophospholipids (Table 2). Metabolic pathway enrichment was also performed but showed no statistically significant enrichment, primarily because many software do not include lipid metabolism in significant detail.

**Table 1.**
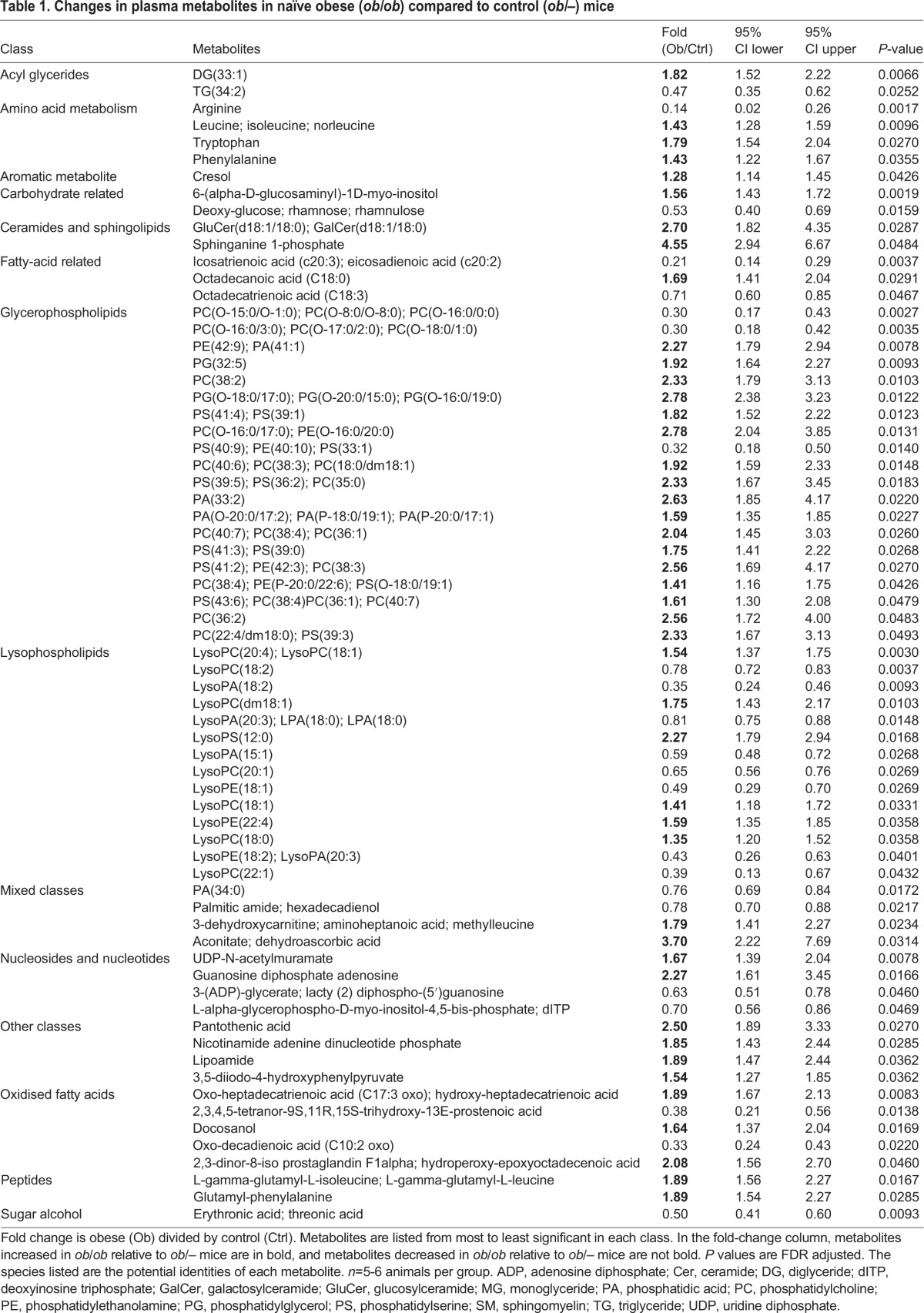
**Changes in plasma metabolites in naïve obese (*ob/ob*) compared to control (*ob/–*) mice**

**Table 2.**
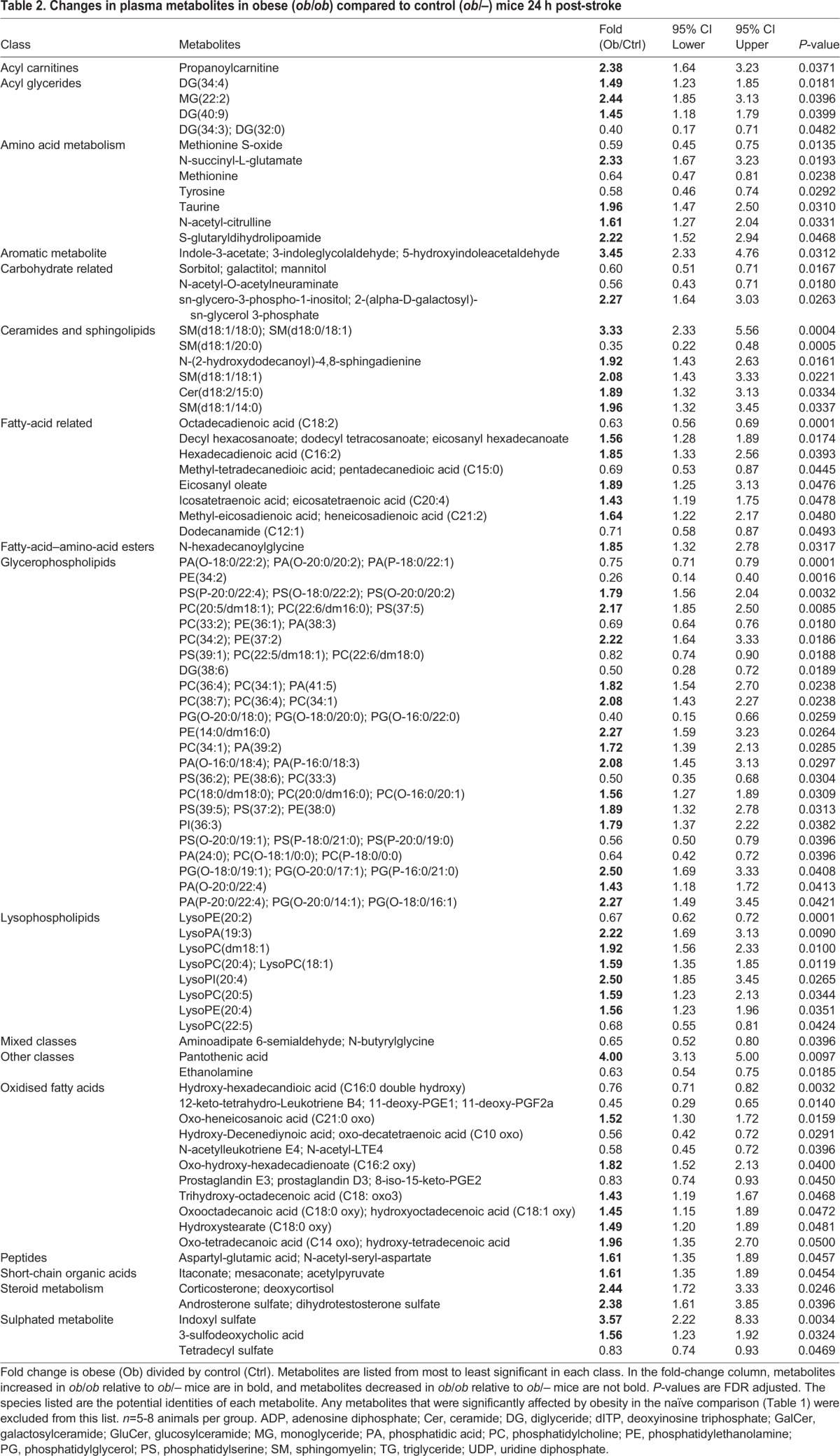
**Changes in plasma metabolites in obese (*ob/ob*) compared to control (*ob/–*) mice 24 h post-stroke**

## DISCUSSION

**Fig. 6.**
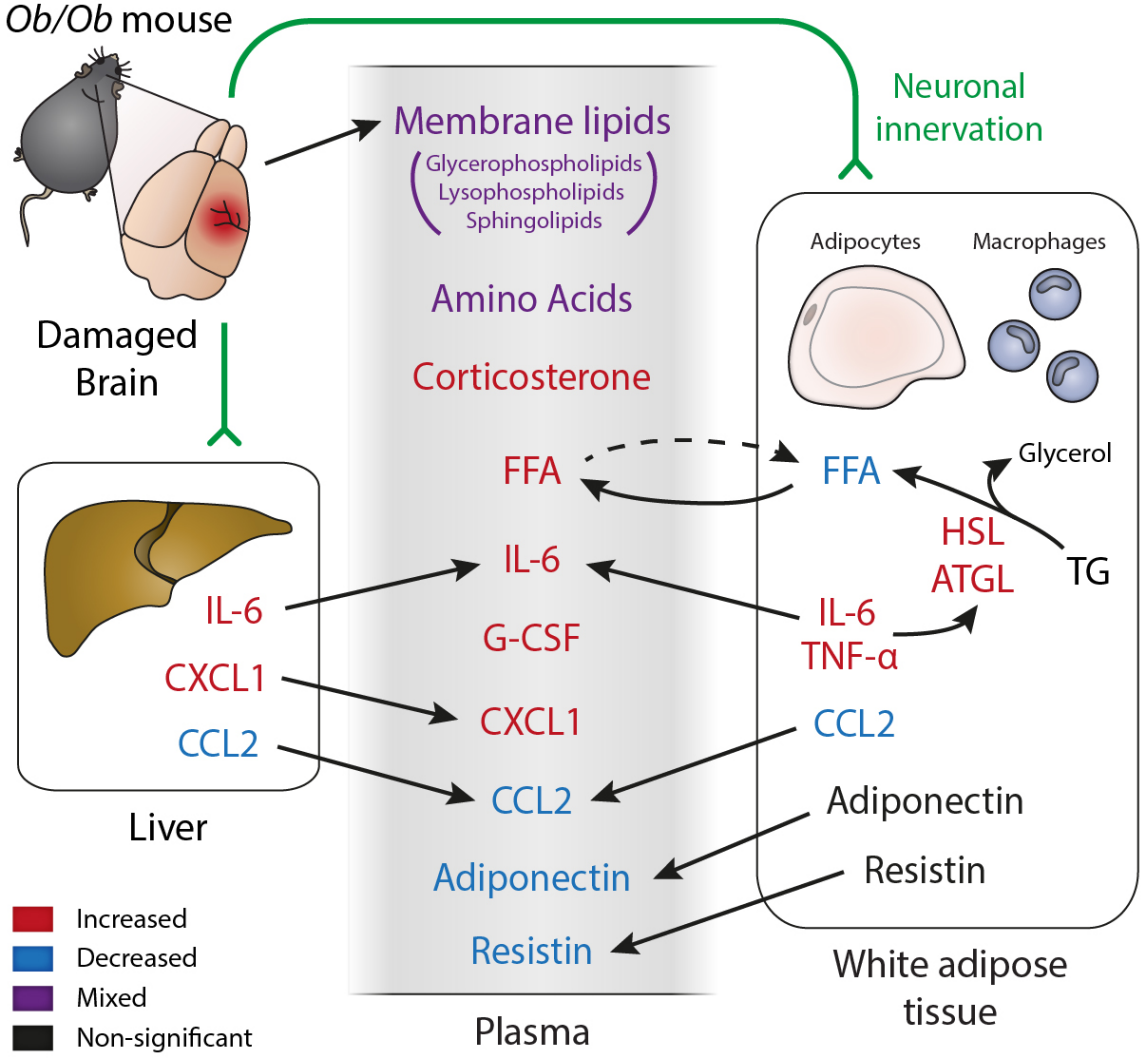
**A summary of the inflammatory and metabolic changes observed in obese *ob/ob* mice acutely (24 h) after stroke, and how we hypothesise they may be related.** Black text indicates no significant effect, red text indicates observations of significant increases, blue text indicates significant decreases and purple text indicates both significant increases and decreases within this category. We hypothesise that acute brain damage caused by hypoxia signals to the liver and adipose tissue either by humoral or neuronal routes. This leads to alterations in expression and release of inflammatory cytokines, which may impact on the metabolic functions of the liver and adipose tissue. For example, pro-inflammatory cytokines can promote lipolysis and promote transport of FFA into the blood. The liver and adipose tissue may also release cytokines into the circulation. Membrane lipids found in the blood may be partially derived from damaged membranes within the CNS.

Stroke is known to affect metabolism in humans, with weight loss commonly reported in stroke patients, where it is associated with poor functional recovery (FOOD Trial Collaboration, 2003; Scherbakov and Doehner, 2011). In this context, the metabolic reserves found in obesity have been suggested to improve tolerance to post-stroke catabolism, therefore improving stroke survival and outcome (Scherbakov et al., 2011). Weight loss is also found in mice within the first 24 h post-stroke, although animals continue to lose weight for a number of days post-stroke (Dirnagl, 2010). However, the mediators of this catabolic drive have not been well characterised, but are hypothesised to include inflammatory mediators such as IL-6 and TNFα (Scherbakov et al., 2011). Previous studies in mice have demonstrated that there is an acute inflammatory response in the periphery after stroke, including in metabolically active tissues such as the liver and adipose tissue (Denes et al., 2010b; Offner et al., 2006; Wang et al., 2011a). Therefore, we characterised the acute (24 h) metabolic response to stroke and potential inflammatory mediators in the context of concurrent obesity (Fig. 6). We show for the first time that the metabolic response to stroke is different in obese compared to control mice. This observation was primarily related to lipid metabolism, which was also altered naïvely in obese animals. The altered metabolic response in obese animals was accompanied by increased cytokine expression in the liver and plasma, and an obese-specific inflammatory response in the adipose tissue.

The initial ischaemic insult in stroke is accompanied by a rapid induction of inflammatory cytokines and chemokines in the blood and peripheral organs (Chapman et al., 2009; Offner et al., 2006). Here, we found an increased plasma response in IL-6, CXCL1 and G-CSF in obese mice after stroke. Increases in plasma CXCL1 have also been observed after stroke in diet-induced obese mice (Maysami et al., 2015). In patients, the magnitude of this IL-6 response correlates positively with stroke severity (Smith et al., 2004, 2006a). Because *ob/ob* mice have worse outcome after experimental stroke, this increased peripheral immune response may be a cause or consequence of their increased ischaemic damage (Haley and Lawrence, 2016b; Mayanagi et al., 2008; McColl et al., 2010; Terao et al., 2008). An exception to the pattern of increased peripheral inflammation in *ob/ob* mice after stroke was the reduction in levels of CCL2 (MCP-1). Plasma CCL2 levels in *ob/ob* mice have been reported to be unaffected by obesity *per se*, but increased after stroke (Terao et al., 2008). However, previous reports in patients suggest that plasma CCL2 levels increase with obesity (Kim et al., 2006), potentially explaining the high basal levels found here, although the reason for the reduction in CCL2 after stoke in obese animals is unclear. Stroke also resulted in an inflammatory response in the fat of obese mice characterised by increased IL-6, TNFα and ICAM-1 expression. A similar pro-inflammatory response has been reported in rats and aged mice after middle cerebral artery occlusion (MCAO), which is associated with deficits in insulin sensitivity (Shin et al., 2015; Wang et al., 2011a). Furthermore, surgical removal of visceral fat in aged mice prior to MCAO reduced lesion volume (Shin et al., 2015). How this protective effect is mediated is unclear, but highlights that the adipose tissue is not just a passive store of fat, but is a highly active endocrine organ. Indeed, the adipose tissue in obese humans becomes a potent source of inflammatory mediators, including IL-6 and IL-8 (the mouse homologue of IL-8 is CXCL1) (Fain, 2010). Therefore, the post-stroke plasma increases in IL-6 and CXCL1 found in obese mice here could be due to increased release from the adipose tissue.

Adipose tissue is also capable of releasing a growing list of factors termed adipokines. Here, we found a decrease in the adipose expression of both resistin and adiponectin in obese mice prior to stroke, as has been previously reported in obesity (Fujinami et al., 2006; Kern et al., 2003). However, we found that stroke resulted in a decrease in plasma resistin and adiponectin in both control and obese mice. Because both resistin and adiponectin are exclusively released by adipocytes in mice (Banerjee and Lazar, 2003; Scherer et al., 1995), this suggests that stroke can alter the release of adipokines from adipose tissue. Alterations in plasma adiponectin and resistin have both been implicated in stroke risk in patients (Efstathiou et al., 2005, 2007), although adipokines more generally could affect stroke outcome by modulating the immune system, vasculature, and energy homeostasis (Guri and Bassaganya-Riera, 2010; Haley and Lawrence, 2016a; Trayhurn and Wood, 2005).

The accumulation of excess fat in obesity is known to affect lipid metabolism; however, we found that stroke triggered a further effect on lipid metabolism in obese animals that was not seen in controls. This was characterised by an increase in plasma FFA, and decrease in epididymal fat FFA at 24 h post-stroke. Plasma FFA concentrations are tightly regulated by hormonal regulation to ensure tissues receive an appropriate supply of fatty acids for use as an energy source. However, fatty acids may also drive pathology, especially when concentrations remain inappropriately high (Frayn et al., 1996). The majority of FFAs in the plasma are derived from lipolysis of triglycerides within adipocytes (Frayn, 2012), suggesting that an increase in lipolysis may underlie this increase in obese mice.

Stroke had differential effects on the expression and phosphorylation of lipolytic enzymes in control and obese mice. The activity of lipolytic enzymes in adipose tissue determines the rate of hormone-stimulated and basal lipolysis. In mice, over 95% of triglyceride hydrolysis in adipocytes is due to the enzymes HSL and ATGL (Schweiger et al., 2006). In order to quickly respond to fluctuating energy requirements, HSL activity is usually mediated by phosphorylation, rather than by a change in protein expression (Anthonsen et al., 1998). Through this regulation, HSL activity is increased pre-prandially by β-adrenergic stimulation, and decreased post-prandially by insulin. After experimental stroke, obese mice had increased HSL expression but no change in Ser563 phosphorylation. Conversely, in control mice, stroke had no effect on HSL expression, but increased Ser563 phosphorylation. This suggests that HSL was stimulated by hormonal exposure in control mice, but was affected by a different mechanism in obese mice.

Rather than being due to hormonal stimulation, increased circulating FFAs found in obese mice post-stroke may be due to increased expression of inflammatory mediators in the adipose tissue. TNFα has been shown to raise plasma FFA levels by acting on the adipose tissue and liver to stimulate basal lipolysis and reduce FFA reuptake (Chen et al., 2009). Pro-inflammatory mediators can increase basal lipolysis in adipocytes via NF-κB and other inflammatory pathways, with the result being an increase in the rate of basal lipolysis independently of HSL phosphorylation (Doerrler et al., 1994; Grisouard et al., 2012; Langin and Arner, 2006). Furthermore, stimulation of lipolysis by the pro-inflammatory endotoxin lipopolysaccharide *in vivo* involves upregulation of HSL expression without an increase in its phosphorylation (Osto et al., 2011). The increase in ATGL expression seen in obese mice also suggests an increase in basal lipolysis, since it is an important mediator of basal lipolysis in rodents (Langin et al., 2005; Zimmermann et al., 2004). Therefore, stroke may trigger an inflammatory response in the adipose tissue of obese mice that results in an increase in basal lipolysis, and thus in increased plasma FFA concentrations. This may be accompanied by a reduction in the clearance of FFAs from the plasma, which has previously been reported in obese patients (McQuaid et al., 2011).

Because control *ob/–* and obese *ob/ob* mice appeared to show different metabolic responses to stroke, particularly in lipid metabolism, we decided to study metabolism at the whole-organism level by applying non-targeted UHPLC-MS metabolomics. In this analysis, obesity *per se* in the absence of stroke affected several classes of metabolites in the plasma of *ob/ob* mice. These changes were not dependent on changes in diet composition because *ob/ob* mice become obese due to hyperphagia. In comparison, metabolomic studies using mice that are obese due to a high-fat diet have the caveat of not being able to differentiate effects occurring as a consequence of diet from those occurring as a consequence of obesity (Duggan and Hittel, 2011). However, a potential caveat of using the obese *ob/ob* mouse in the current study is their lack of the adipokine leptin, which is known to modulate energy balance and the immune system (Trayhurn and Wood, 2005; Zhang et al., 1994).

A large number of the metabolites detected as being significantly affected by obesity in mice here were lipid related, with lipid species overall being increased in obese *ob/ob* mice, as expected. The largest proportion of these lipids was membrane-associated, including glycerophospholipids, lysophospholipids and sphingolipids. Lipidomics in humans has demonstrated that obesity generally increases the abundance of lipid species, and certain species have been found to correlate with body mass index (BMI), including acyl glycerides, phosphatidylethanolamines, phosphatidylcholines and lysophospholipid species (Graessler et al., 2009; Oberbach et al., 2011; Wallace et al., 2014). Furthermore, several of the lysophospholipid species that increased in obese animals in the present study [specifically lysophosphatidylcholines (lysoPCs) 18:0, 18:1 and 18:2] have been correlated with measures of insulin resistance and inflammation in patients (Wallace et al., 2014). Mice that develop obesity due to being fed a high-fat diet also showed increases in fatty acids, phosphatidylcholines and lysophospholipid species (Kim et al., 2011; Sampey et al., 2012). Two specific metabolite or metabolite classes that require further investigation are sphinganine-1-phosphate (4-fold higher in naïve *ob/ob* mice) metabolites and the metabolite class of plasmalogens. The sphinganine-1-phosphate-related metabolite sphingosine-1-phosphate has been implicated in poor outcome for ischemic stroke (Ichijo et al., 2015; Maceyka et al., 2013; Moon et al., 2015) and sphingolipid metabolism before and after stroke is therefore worthy of investigation. Plasmalogens are identifiable by their ether linkage at the sn-1 position, and can make up 20% of all glycerophosphocholines in the brain (Farooqui and Horrocks, 2001). Plasmalogens have numerous important roles in the central nervous system (CNS), including protecting against reactive oxygen species damage; thus, the observation that specific metabolites both increased and decreased could be relevant for subsequent stroke outcome.

Beyond effects on lipid metabolism, changes in other metabolites in *ob/ob* mice were suggestive of health complications usually associated with obesity. Obese mice had increases in the amino acids leucine and phenylalanine. In patients, these were found to predict future diabetes and correlate positively with BMI (Gralka et al., 2015; Ho et al., 2016; Shah et al., 2012; Wang et al., 2011b). Obese *ob/ob* mice also had reduced plasma arginine, being the most greatly reduced (7-fold) metabolite species detected. Similar findings of reduced plasma arginine have been made in genetically obese Zucker rats (Serkova et al., 2006). Reductions in plasma arginine have been implicated in the development of cardiovascular disease (Shah et al., 2012; Tang and Wang, 2009), potentially because arginine deficiency can result in endothelial dysfunction and decreased nitric oxide availability (Morris, 2012). Arginine and associated metabolic products have also been demonstrated to be acutely elevated post-stroke, so the potential impact of reduced arginine found in obese subjects on subsequent stroke outcome requires further study (Molnar et al., 2014). Furthermore, in obese mice a reduction in a carbohydrate deoxy-glucose species was observed, which may correspond to 1,5-anhydroglucitol, a marker of prolonged hyperglycaemia and predictor of future diabetes (Dungan et al., 2006). Overall, the metabolite profile of *ob/ob* mice shows biomarkers not only of obesity, but also aspects of the metabolic syndrome, including insulin resistance, dyslipidaemia and cardiovascular disease. This suggests that the obese *ob/ob* mice possess a spectrum of risk factors for stroke similar to those seen in obese patients.

Metabolomic analysis of metabolites in the plasma at 24 h post-stroke was performed in both obese *ob/ob* and control mice. A comparison of these two profiles found 83 unique metabolites to be significantly affected by obesity after stroke. As this list excluded metabolites detected as being affected by obesity in the absence of stroke, this list entails the obese-specific metabolic response to stroke. A large number of these species were lipid-related, including several classes of membrane lipids. Further changes in glycerophospholipids and lysophospholipids were found in addition to those altered by obese mice in the absence of stroke. LysoPC(20:4) was increased in obese mice relative to control mice after stroke, and is a reported biomarker of ischaemic stroke in patients (Jové et al., 2015; Zhu et al., 2015). Stroke also affected long-chain sphingomyelin (SM) sphingolipids in obese animals. Two species that were increased in obese mice after stroke, SM(d18:1/18:0) and SM(d18:1/18:1), were found to be specific markers of acute traumatic and ischaemic brain injury in mice (Sheth et al., 2015). Sphingomyelin lipids also correlate positively with plasma IL-8 and TNFα in obese subjects (Wallace et al., 2014) and are implicated in the development of cardiovascular disease due to their promotion of pro-inflammatory signalling pathways (Nixon, 2009; Sun et al., 2016). Therefore, the upregulation of sphingomyelins in response to stroke was specific to obese mice and may be a marker of post-stroke inflammation or greater damage in obese mice. Alternatively, sphingolipid species may worsen outcome by activating pro-inflammatory signalling pathways (Sun et al., 2016). The increase in oxidised forms of fatty acids similar to those found abundantly in obese *ob/ob* adipose tissue (long-chain fatty acids, C_14_ to C_18_) suggests that fatty acids liberated by lipolysis in obese mice after stroke are undergoing incomplete β-oxidation to be used as an energy source.

The differences in the metabolic response to stroke between obese and control animals were not restricted to lipid species, with several classes of non-lipid metabolites also significantly affected. Corticosterone was significantly higher in obese animals after stroke compared with control (*ob/−*) mice post-stroke, suggestive of increased HPA axis activation. Activation of the HPA axis results in the release of catecholamines and glucocorticoids that can directly stimulate lipolysis, and thus affect plasma lipid concentrations. Amino acids were also affected, with obese animals showing reductions in tyrosine and methionine, and an increase in taurine. Similar changes in plasma amino acids have been reported as biomarkers for ischaemic stroke in both patients and rodents, although the directions of these changes vary (Gao et al., 2013; Kimberly et al., 2013; Liu et al., 2015; Zhu et al., 2015). Because the extent of these changes can correlate with the severity of ischaemic damage (Kimberly et al., 2013), the difference between obese and control animals found here could be due to increased ischaemic damage in obese mice.

Weight loss for several days after experimental stroke is common in rodents, and often correlates with stroke severity (Dirnagl, 2010). In the current study, absolute weight loss was similar between obese and control mice, although obese mice lost less weight as a percentage of baseline weight. There was also no correlation between either measure of weight loss and ischaemic damage. However, it is unclear from what compartment this weight is being lost at this acute time point. For example, weight may be lost not only from adipose tissue, but potentially from glycogen stores, muscle or other lean stores. Therefore, weight loss at this acute time point may not reflect long-term changes in metabolism. In order to assess the acute metabolic response to stroke, we selected an acute time point that is less likely to be affected by other complications of stroke such as inactivity and motor deficits. However, future work is required to establish whether acute changes in metabolism translate into longer-term changes in metabolism and outcome.

In the present study, obesity affected the metabolic response to stroke, characterised by an increase in plasma FFAs, likely due to an increase in basal lipolysis. Metabolomics revealed that the obese-specific response to stroke featured changes in various lipid species, including acyl glycerides, sphingolipids, glycerophospholipids, lysophospholipids and fatty-acid derivatives. This altered metabolic response to stroke may be driven by or may drive a post-stroke inflammatory response in the adipose tissue of obese mice that was not seen in control mice. The adipose tissue has previously undergone little investigation in the response to stroke, although may affect outcome due its ability to release adipokines and pro-inflammatory cytokines. These differences in the metabolic and inflammatory responses to stroke in obese mice highlights the importance of considering comorbidities in preclinical studies, especially when considering the usefulness of biomarkers of stroke. It will also be important to consider how obesity affects the loss of muscle mass after stroke, as this will be an important determinant of functional recovery, although pro-inflammatory cytokines are known to promote catabolism that can lead to sarcopenia (Kotler, 2000). Overall, whether these results support the hypothesis that obesity protects against post-stroke weight loss will depend on whether these acute changes in lipolysis, inflammation and metabolism translate into longer-term effects on recovery or outcome.

## MATERIALS AND METHODS

### Mice

Experiments were performed on 16- to 19-week-old male obese *ob/ob* (C57BL/6OlaHsd-Lep^ob^, *n*=26) or male heterozygote *ob/–* control (*n*=26; Harlan-Olac, UK) mice. Body weight at the time of surgery was 32.5±5.7 g for control (*ob/–*) and 54.1±6.0 g for obese (*ob/ob*). All mice were housed in standard conditions (temperature of 20±2°C; humidity, 55±5%; 12-h light/12-h dark cycle with lights on at 08:00 h) and were given *ad libitum* access to a standard laboratory diet (RM1, Special Diet Services, UK) and water. All experimental procedures using animals were conducted in accordance with the United Kingdom Animals (Scientific Procedures) Act, 1986 and approved by the Home Office and the local Animal Ethical Review Group, University of Manchester.

### Focal cerebral ischaemia

Focal cerebral ischaemia was induced in control (*ob/–*) and obese (*ob/ob*) mice by transient (20 min) MCAO. We have previously published that this approach and duration of occlusion leads to worse outcome in *ob/ob* mice, and this was confirmed in a subset of animals in this study (*n*=6 per group) (Haley and Lawrence, 2016b). Briefly, under isoflurane anaesthesia, the carotid arteries were exposed and a silicone-coated filament (coating 210 μm in diameter and ≥5 mm length, Doccol, USA) was introduced into the external carotid artery and advanced along the internal carotid artery until occluding the origin of the middle cerebral artery. Successful MCAO was confirmed by a reduction in cerebral blood flow of at least 80% as measured using laser-Doppler (Moor Instruments, UK). After 20 min, the filament was withdrawn to establish reperfusion and the wound sutured. During surgery, core body temperature was monitored using a rectal probe and maintained at 37°C using a homeothermic blanket (Harvard Apparatus, Kent, UK). Animals were allowed to recover and, 24 h later, were weighed and re-anesthetised. Cardiac blood was collected (with sodium citrate to prevent clotting), spun at 1400 ***g*** for 10 min at 4°C and aliquots of plasma were immediately frozen. Samples of liver and epididymal fat pads were also taken for homogenisation in buffer (50 mM Tris-HCl, 150 mM NaCl, 5 mM CaCl_2_, 0.02% NaN_3_, 1% Triton X-100). Naïve animals (*n*=10 per genotype) were treated identically, but underwent no experimental ischaemia. All animals were randomly assigned to a treatment group (cerebral ischaemia versus naïve). In each of the control *ob/–* and obese *ob/ob* stroke groups, 16 animals underwent surgery, with 2 animals excluded from each group due to subarachnoid haemorrhage or death based on pre-established criteria (final *n*=14 per genotype).

### Quantification of cytokines

Concentrations of cytokines were measured in tissue homogenates or plasma samples by cytometric bead array (CBA) as defined by the manufacturer's instructions, with Flex Sets used for IL-6, TNFα, CCL2, CXCL1 and G-CSF (BD Biosciences). ICAM-1, adiponectin and resistin were measured in epididymal fat homogenates or plasma samples by ELISA according to the manufacturer's instructions (DuoSet, R&D Systems).

### Glucose, FFA, glycerol and triglyceride measurement

Blood glucose was measured in tail vein blood samples taken immediately prior to experimental ischaemia or at time of sacrifice using a hand-held glucose monitor (Accu-Chek Aviva, Roche, UK). FFAs and glycerol were quantified in cardiac plasma samples and epididymal adipose tissue homogenates, and triglycerides and glycerol were quantified in liver homogenates, as per the manufacturer's instructions using commercially available kits (Zen Bio, USA).

### Adipose tissue HSL and ATGL assays

Expression of HSL, its serine-563-phosphorylated form (HSL Ser563) and ATGL were measured in epididymal fat pad homogenates by western blot. Antibodies for immunoblotting were as follows: anti-HSL (1:2000, Cell Signaling Technology, USA), anti-HSL Ser563 (1:500, Cell Signaling Technology), anti-ATGL (1:2000, Cell Signaling Technology) and anti-β-actin (1:5000, Sigma). Epididymal adipose tissue homogenates (25 µg total protein per well) were separated by SDS page, and proteins transferred to a polyvinylidene fluoride membrane. Membranes were then blocked [5% milk and 0.1% TWEEN-20 in PBS (PBS-T)], and incubated at 4°C overnight in primary antibodies diluted in 1% BSA in PBS-T. After being incubated with HRP-conjugated secondary antibodies (1:500, Wako Chemicals) in 5% milk in PBS-T, blots were developed using an Enhanced Chemiluminescent Detection Kit (GE Healthcare). Digital images of protein bands were acquired (ImageQuant 350, GE Healthcare, UK) and semi-quantitative analysis of protein content performed by densitometry using ImageQuant TL software (GE Healthcare, UK), with values normalised to β-actin as a loading control.

### Non-targeted metabolomics

Mouse plasma was taken from control *ob/–* and obese *ob/ob* mice without previous treatment (naïve, *n*=5-6/group) and at 24 h post-stroke (*n*=6-8/group) and prepared as previously described (Dunn et al., 2011b). Plasma was thawed on ice and deproteinised by addition of 300 μl of methanol to 100 μl of plasma followed by vortex mixing (15 s) and centrifugation (13,365 ***g*** for 15 min). The supernatants (300 μl) were transferred to 2.0 ml centrifuge tubes and vacuum dried. Prior to analysis the dried extracts was reconstituted in 100 μl of 50/50 water/methanol. Additionally, pooled QC samples were prepared by pooling 75 μl aliquots of each biological sample; subsequently, 100 μl aliquots were deproteinised, dried and reconstituted as described above.

Samples were analysed twice (in positive ion and negative ion modes), applying an Accela UHPLC system interfaced to an electrospray LTQ-Orbitrap Velos hybrid mass spectrometer (Thermo Fisher Scientific, Hemel Hempstead, UK). Chromatographic separations were performed on a Hypersil GOLD column (100×2.1 mm, 1.9 μm; Thermo Fisher Scientific, Runcorn, UK) operating at a column temperature of 50°C. A binary gradient elution was applied at a flow rate of 400 μl min^−1^ with 2 solvents: solvent A=0.1% formic acid in water (vol/vol) and solvent B=0.1% formic acid in methanol (vol/vol). Solvent A was held at 100% for 0.5 min followed by an increase to 100% solvent B over 15.5 min, which was then held at 100% solvent B for a further 5 min. A step change to 100% solvent A was performed at 20.5 min and then held at 100% solvent A to equilibrate for 1.5 min. All column eluent was transferred to the mass spectrometer and full-scan profiling data were acquired in the *m/z* range 100-1000 in the Orbitrap mass analyser (mass resolution 30,000; full width half maximum at *m/z*=400). The source and ion transfer parameters applied were as follows: source heater=200°C, sheath gas=50 (arbitrary units), aux gas=15 (arbitrary units), capillary temperature=300°C, ISpray voltage=4 kV (positive ion mode) and 3 kV (negative ion mode), s lens=65% and AGC=5×10^5^.

Raw instrument data were converted to netCDF file format with FileConverter software available in XCalibur (Thermo Fisher Scientific, Bremen, Germany). Deconvolution was performed using the freely available XCMS software as described previously (Dunn et al., 2011b; Smith et al., 2006b). Data were exported from XCMS as a .csv file for further data analysis. The quality of data was assessed by applying QC data as previously described (Smith et al., 2006b), with all metabolite features with an RSD >20% or with greater than 30% missing values for QC samples being removed from the dataset prior to data analysis. Metabolite annotation was performed by applying the PUTMEDID_LCMS workflow as previously described (Brown et al., 2011). All metabolite annotations are reported at level 2 according to MSI reporting standards (Sumner et al., 2007). The processed data was analysed in R with the univariate *t*-test applying the Benjamini–Hochberg procedure for FDR calculation (*q*<0.05) after normalisation to total peak area. The fold change was calculated by applying the median response per class with 95% confidence limits. The processed data were also analysed by applying unsupervised PCA, and pairwise comparisons of classes by applying supervised PLS-DA; both of these analyses were performed in the open-access software MetaboAnalyst (Xia et al., 2012). Metabolites were manually clustered into classes defining similar chemical structure or metabolic pathway to identify biologically relevant and robust metabolic changes.

### Statistical analyses

All *ex vivo* analyses were performed blinded to treatment (naïve or MCAO) and genotype (control or obese). Sample sizes were determined by power calculation (α=0.05, β=0.2) of our previous data. Statistical analyses for all non-metabolomic data were performed using GraphPad software (GraphPad Software Inc., La Jolla, CA, USA). Data was tested for equal variance using the Brown–Forsythe and Bartlett's tests, and appropriate transformations applied if necessary. Two-way ANOVA was used for comparisons by genotype and by treatment followed by a Šídák correction for multiple comparisons, and a *t*-test was used for two groups. Correlations between body weight loss and ischaemic damage were assessed by Pearson correlation coefficient. All data (apart from metabolomics) are represented as means±standard error of the mean (s.e.m). *P*<0.05 was considered significant.
